# A Framework for Developing Intake and Use Guidance for Dietary Supplement Bioactives^☆^

**DOI:** 10.1016/j.advnut.2026.100623

**Published:** 2026-03-25

**Authors:** Janet A Novotny, Johanna Dwyer, Mario G Ferruzzi, Bill Gurley, Tasija Karosas, Colin D Kay, Esther Myers, Andrew Shao, Connie M Weaver

**Affiliations:** 1 Beltsville Human Nutrition Research Center, Agriculture Research Center, USDA, Beltsville, MD, United States; 2 Department of Medicine, Tufts University School of Medicine and Jean Mayer USDA Human Nutrition Research Center on Aging at Tufts University, Tufts Medical Center, Boston, MA, United States; 3 College of Agriculture and Life Sciences, Virginia Tech, Blacksburg, VA, United States; 4 National Center for Natural Products Research, School of Pharmacy, University of Mississippi, University, MS, United States; 5 The International Life Sciences Institute, Washington, DC, United States; 6 Arkansas Children’s Nutrition Center, University of Arkansas for Medical Sciences, Little Rock, AR, United States; 7 International Affiliate of the Academy of Nutrition and Dietetics, United States; 8 EF Myers Consulting, Inc., Trenton, IL, United States; 9 Niagen Bioscience, Los Angeles, CA, United States; 10 School of Exercise and Nutritional Sciences, San Diego State University, San Diego, CA, United States

**Keywords:** bioactives, bioactive substances, dietary supplements, recommendations, dietary ingredients, risk

## Abstract

Dietary bioactives, which are commonly found in food, beverages, and dietary supplements, include nonessential food components that can support health beyond preventing nutrient deficiencies. Despite their known health benefits and potential for adverse effects, there is limited guidance on appropriate intake levels, safety, and efficacy of these ingredients, particularly those present in dietary supplements. This lack of guidance can be challenging for individuals attempting to make informed decisions about products marketed for health benefits. A previously published perspective proposed a 4-step framework for developing quantitative intake recommendations for bioactive constituents in food. This framework addressed bioactive characterization, safety evaluation, efficacy assessment, and how to develop intake levels from foods. However, it did not account for the unique considerations of bioactives consumed in dietary supplement form. The current paper builds on the original framework by addressing the specific factors that must be considered when developing intake guidance for bioactive constituents found in dietary supplements. These include differences in dose, matrix, purity, bioavailability, and intended use. In doing so, this paper aims to advance the development of evidence-based recommendations for supplements, while also highlighting the fundamental distinctions between bioactive constituents delivered through food and diet compared with those delivered through dietary supplements.

**Statement of significance**

A framework is presented to provide a stepwise approach to evaluate and synthesize evidence into evidence-based recommendations for the safe use of bioactive constituents in dietary supplements. The framework has broad use across the research community, industry, and the healthcare landscape as a reference for development of use guidance, for impactful research design, and for dietary supplement production.

## Introduction

Foods, beverages, and dietary supplements often contain bioactive constituents that include essential nutrients and non-nutrients that may have both beneficial and adverse effects on health [1]. Although consumers have access to ample information about the amounts of essential nutrients in foods that are required for health, there is limited guidance on the safety and efficacy of other bioactive compounds found in food and dietary supplements. This lack of guidance makes it difficult for consumers to make informed decisions, especially when choosing products intended to support their health. Given that bioactive constituent intake is the sum of ingestion from all sources (both food and dietary supplements, and some nutrient-containing medications), the lack of guidance for non-nutrient bioactive–containing dietary supplement products limits the ability to evaluate underconsumption and overconsumption among dietary supplement users. For the purposes of this paper, the authors use the term non-nutrient bioactive–containing dietary supplements to refer broadly to products ranging from extracted purified compounds, such as lutein supplements, to botanical preparations, such as ginger root supplements containing gingerols.

At present, a framework for developing evidence-based guidance on which consumers can make decisions about bioactive-containing dietary supplement use based on efficacy and safety criteria is lacking. Therefore, it is useful to describe the characteristics of a framework for developing recommendations for the use of dietary supplement products containing nonessential bioactives. Such a framework will facilitate the development of guidance that ensures dietary supplement users consume the total amounts of bioactive constituents in their diets that are safe and sufficient to achieve the desired health-related effects on function or health outcomes.

The need for such guidance has grown since the use of these products is now widespread in the United States after the implementation of the Dietary Supplement Health and Education Act (DSHEA) [2] over a quarter of a century ago. Fifty-seven percent of respondents to the 2017–2018 NHANES reported supplement use in the past 30 d [3], and in the 2023 Council for Responsible Nutrition survey on supplement use, 74% of adults use supplements, with 55% considering themselves to be “regular” supplement users [4]. Moreover, the number of dietary supplement products currently on the market exceeds 75,000 [5]. Some of these products contain well-characterized essential nutrients with extensive and well-validated information about their safety and efficacy, based on sound science, available in the public domain. However, other products contain many non-nutrient bioactive constituents/ingredients in amounts and forms that have heretofore been limited in exposure from consumption of typically available food sources, and for which little data on their safety, efficacy, and quality are available in the peer-reviewed literature. Moreover, studies of their efficacy may not be available in the public domain. These gaps in knowledge make it difficult for those who seek guidance to readily find helpful evidence-based advice [6].

This paper provides a pathway to evaluate and synthesize evidence into evidence-based recommendations for the safe use of bioactive-containing dietary supplements to achieve claimed metabolic and health-related benefits. The National Academy of Sciences, Engineering and Medicine (NASEM) has established methods to develop recommendations for the consumption of essential nutrients known as dietary reference intakes (DRI). However, there are differences between essential nutrients and non-nutrient constituents such that the traditional DRI approach does not apply to non-nutrients, as shown in Table 1. Those differences necessitate the development of different approaches for arriving at recommendations for intakes of these different types of constituents for individuals who wish to use them. Some of the challenges for use of the traditional DRI-like approach for non-nutrient bioactives include general lack of information about the constituents, lack of sound intake-response data over a range of intake levels, lack of a unique indicator of response for most nonessential nutrients, potential effects that are often small and slow to develop and thus cannot be assessed over a short period of time in clinical studies, nonunique functions of nonessential bioactives, and lack of funding and incentives for establishing a firmer scientific base for recommended intakes [7]. NASEM committees have proposed guiding principles for incorporating chronic disease endpoints into setting DRI values [[7], [8], [9]] and criteria for establishing evidence for amounts of non-nutrient constituents that are needed to affect validated markers of health outcomes. However, intake levels for safe amounts of non-nutrient bioactive constituents with specific impacts on unvalidated surrogate markers of health outcomes were not addressed.

**TABLE 1:** Key differences between nutrients and non-nutrient bioactives that influence the development of intake guidance.

Characteristic	Nutrients	Non-nutrient bioactives
Prevalence	Limited in number	Countless
Statutory definition?	Yes	No
Essential for life?	Yes	No
Functions, mechanisms of action, and targets known?	Yes	Only partially for some
Chemical structures known?	Yes	Only for some
Target group	Everyone	Individuals at risk for a particular health condition or interested in health maintenance and promotion
Efficacy biomarkers widely accepted and agreed on?	Yes	No
Evidence base	Large evidence base for safety and efficacy	Limited and incomplete
Adequate intakes known?	Yes	No
Excessive intakes known?	Yes	No
Intakes to reduce chronic disease known?	For some (sodium, potassium)	Possibly for a few (dietary fiber)
Authoritative intake guidance?	Yes	Very limited

To address the need for a guide for the development of intake recommendations for non-nutrient bioactives, one that differs from the traditional DRI process [6], a position paper was recently published presenting a step-by-step method for translating available evidence into quantitative intake recommendations for bioactive constituents in food [10]. The framework presents a 4-step process for evaluating evidence related to characterization of a given bioactive constituent in food, evaluation of safety, review of efficacy data, and development of quantified intake recommendations. However, the framework was solely directed toward food bioactives and excluded the unique considerations for bioactive constituents provided in dietary supplement form [11].

The primary difference between ingestion of dietary supplements compared with ingestion of food is the greater risk of exposure to high concentrations of specific bioactive constituents of unknown safety when consumed in concentrated form [11]. The greater potential exposure risk stems from differences between food and dietary supplements with respect to matrix, purity, dose, and other conditions of use. In the case of food, matrix, volume, and energy can serve to limit intake, whereas dietary supplements, which are often formulated with extracts or purified bioactives, tend to contain highly concentrated ingredients within matrices that can allow for relatively large acute doses of the active compounds and other components. Therefore, the development of guidance for the use of non-nutrient bioactive–containing dietary supplements requires considerations distinct from those applied to food-based bioactives.

The framework presented here has a broad use across the research community, industry, and the healthcare landscape, as described in Table 2. It is intended to guide professional societies and expert groups in developing intake and use guidance that can subsequently be used by health professionals, such as doctors, nurses, and dietitians, to advise patients, as well as by individuals who use supplements [11]. It can also serve as a resource for expert groups to develop guidance that summarizes the evidence to clearly communicate the safe and effective use of these compounds directly to consumers. In addition to guiding clinical practice and improving consumer knowledge, the framework highlights best practices for research and industry, while addressing the unique challenges of evaluating safe and effective inclusion of bioactive components within dietary supplements. Identifying these challenges will facilitate critical appraisal of research proposals and the body of research for either scoping reviews or systematic reviews. Within the industry, these guidelines will help establish a clear and efficient communication process to ensure that all project needs are understood and met, resulting in products that adhere to the highest safety standards.

**TABLE 2:** Audience and use case for dietary supplements guidance framework.

Audience	Use case
Expert groups	1) To create guidance for physicians and other healthcare professionals to support evidence-based recommendations on supplement use and intake ranges, with the goal of improving patient health outcomes. 2) To create guidance for industry, offering a structured overview of the evidence base for their products to inform development and responsible innovation. 3) To create guidance for consumers to help them understand whether the supplements they are taking are both safe and efficacious.

Researchers and academics	To review existing research and design new studies or identify gaps in current knowledge, resulting in the generation of more data.

Industry professionals	To establish a clear and efficient communication process within the industry to ensure that all project needs are understood and met, resulting in a product that adheres to the highest safety standards.

Evaluators	To support grant writers, manuscript reviewers, and authors to ensure all necessary information is present within the proposal being reviewed.

Regulators/policy makers	To establish alignment on guidance for the substantiation of safety and efficacy of non-nutrient bioactives for inclusion in dietary supplements.

## Overview

Dietary supplements are defined by Congress in DSHEA as products for oral ingestion that contain a dietary ingredient intended to supplement the diet and are labeled as a dietary supplement. “Dietary ingredients” include constituents such as vitamins, minerals, herbs or other botanicals, amino acids, and dietary substances in the food supply, such as enzymes or live microbials (probiotics), and metabolites, concentrates, constituents, extracts, or combinations of “dietary ingredients” [2].

Dietary supplements are *products* that vary greatly in their composition, product form, and conditions of use. They can consist of many ingredients, including purified compounds (extracted or synthesized), combinations of purified compounds, extracts from botanicals, or minimally or highly processed plant components. They also typically include excipients (inactive ingredients that assist with processing and/or stabilizing the dietary supplement) and/or a matrix. In most cases, the supplement provides a concentrated dose of compounds, in comparison to food in natural form, and in a matrix usually distinct from that of conventional foods. Other conditions of use for a dietary supplement that are distinct include recommended frequency and timing, and whether use is in a fed compared with fasted state. The supplement itself is a marketed product, whereas the ingredients are the components of the dietary supplement.

There is no statutory definition of the term bioactives. Thus, in this context, the definition of bioactives is referred to in the same way as that used for the framework for food bioactives [10], which is the NIH Office of Dietary Supplements working definition of bioactives as “constituents in foods or dietary supplements, other than those needed to meet basic human nutritional needs that are responsible for changes in health status” [12].

**FIGURE 1:**
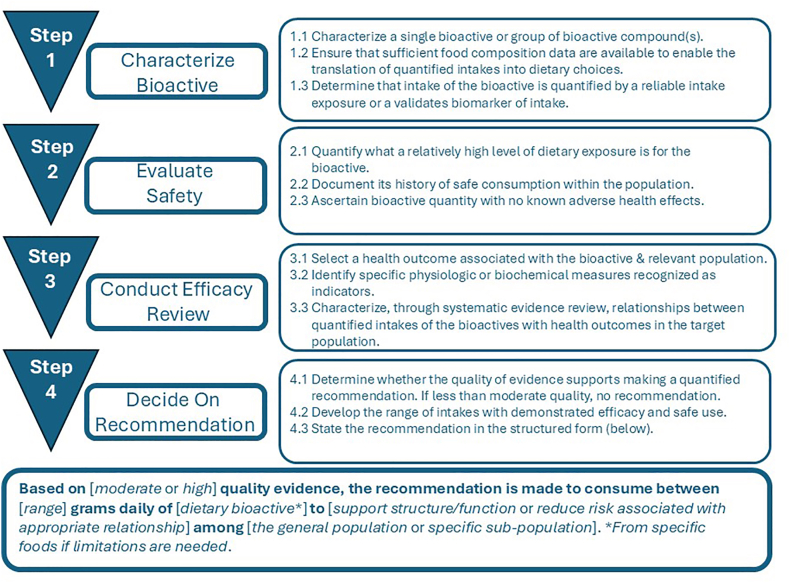
Step-by-step method for translating available evidence into quantitative intake recommendations for bioactive constituents in food [10].

This framework addresses key aspects for development of guidance for use of non-nutrient bioactive–containing dietary supplements in conjunction with that developed by Yates et al. [10] for foods. Although the Yates et al. [10] framework serves as a model for food-based bioactives, the approach presented here extends its application to dietary supplements by incorporating additional considerations unique to these products (Figure 1). The goal of expanding this framework from food bioactives alone is to ensure that total intakes (e.g., total exposure from food, supplements, and medications) are accounted for, for individuals who use bioactive-containing dietary supplements. Although this framework is focused on the specific regulatory environments of the United States and Canada, it may provide some useful guidance for other countries as well. However, an international framework for bioactives contained in finished products (i.e., in supplement form) would be difficult to develop at present because of the lack of harmonization among countries on classification, regulation of products, and types of evidence needed to demonstrate the health benefits of dietary supplements [6,11,12].

Once the body of evidence is sufficient to suggest a possible health benefit, steps for developing guidance for use are as follows: (step 1) define, authenticate, and characterize bioactives included in the dietary supplement, as appropriate for the supplement type, (step 2) evaluate safety, (step 3) conduct efficacy review, and (step 4) decide on the recommendation. When possible, step 4 should include dosage ranges associated with specific benefits/outcome measures that would align with regulatory requirements, with a summary of the rationale for the decision. Analogous steps for food bioactives are described in detail in the paper by Yates et al. [10], and perspectives for extending that original framework are shown below to address dietary supplements.

## Adaptation of the Food-Based Model to Bioactives in Dietary Supplements

The adaptations made in each step will be presented separately.

## Step 1: Define, Authenticate, and Characterize Bioactives in the Dietary Supplement

**FIGURE 2:**
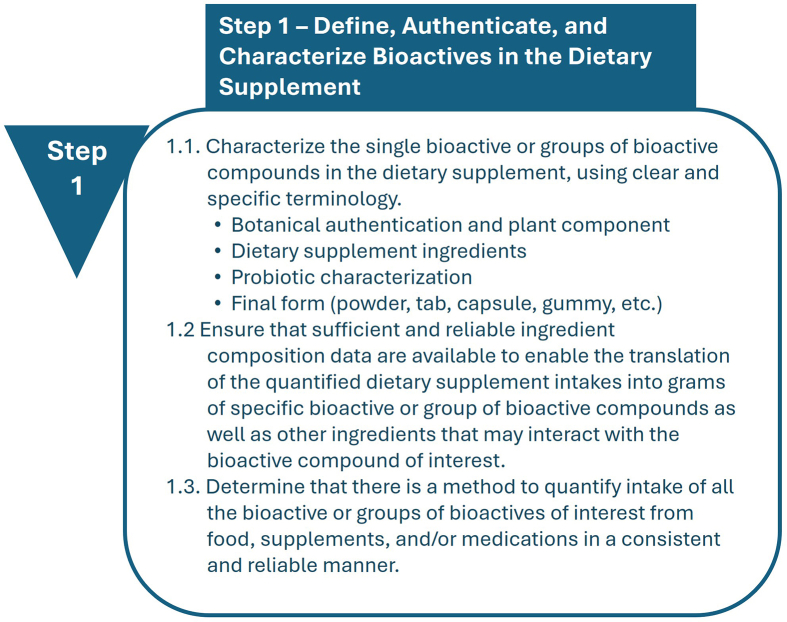
Steps for characterizing dietary supplement bioactive(s) and features of a dietary supplement when initiating a review for safety, efficacy, and development of use guidance.

Clear and complete characterization of the dietary supplement is necessary for authentication and confirmation of safety, for evaluation of efficacy, and for articulation of a recommendation for dietary supplement use (Figure 2). Characterization of food bioactives is addressed in the paper by Yates et al. [10], and characterization of natural products was addressed by Sorkin et al. [13], and many of those principles apply to non-nutrient bioactive–containing dietary supplements as well. Below are provided additional considerations related to dietary supplements.

### Botanical authentication and form

Botanically derived supplements should be characterized and clearly articulated during data review and development of a recommendation. Multiple methods may be needed to authenticate and completely characterize a botanical ingredient, including both macroscopic and microscopic evaluation of morphological characteristics of plant material, genetic assessment, and chemical identification. Each plant source should be authenticated (meaning definitive determination provided of the genus/species), dosage form performance should be assessed (details mentioned later), the plant part used should also be declared (e.g., root, leaf, stems, flowers, and berries), and when available, geographic information should be shared. This information can likely be obtained by the supplier. In the case of supplements containing botanical ingredients, physical and chemical processing (heat treatment, method of storage, extraction solvents, standardization protocols, etc.) will affect the final product. Botanical extracts are complex mixtures containing numerous phytochemicals, with their composition depending on the extraction process and solvents used. This process largely determines the physicochemical properties of the phytochemicals, such as lipophilicity, water solubility, and bioaccessibility, which end up in the final product. Thus, characterization only of the starting material is insufficient, and the final product should be thoroughly characterized as well, including stability of the final product during storage.

Because growth conditions and processing methods alter the chemical composition of the final product, it is common to report quantities of *marker compounds*. Marker compounds are 1 or more phytoconstituents that occur naturally in the botanical material and that are selected for special attention by a researcher or manufacturer. These are often the biologically active target compounds or compounds unique to the botanical. The levels of the marker compounds are quantitatively determined in both raw materials and products and are used as a guide for the manufacture of the product. Data from methods such as UV/VIS spectroscopy, mass spectrometry, and nuclear magnetic resonance are useful for confirming identification and for measuring marker compounds. Data from nontargeted metabolomics (very broad assessment of small molecules) combined with chemometrics (multivariate statistical methods for analyzing large datasets) are also useful for chemical authentication and for monitoring batch-to-batch variation or variation due to other environmental or geographical origins of the same botanical.

### Dietary supplement ingredients

All added ingredients must be listed on dietary supplement labels. All known active ingredients in a dietary supplement must appear in the Supplement Facts box and should be considered in use-related guidance, as they may affect safety and efficacy assessment. Furthermore, the chemical form of the ingredients or standardized compounds should be described as unambiguously as possible. Although the actual ingredients in many dietary supplements are in accordance with the product labels, there are documented instances in which the product ingredients differ from the label claims [14]; thus, confirmation is important. Furthermore, active ingredient stability for the intended conditions of product storage and use must be determined over the stated shelf life, either for research or for health benefits. It should be noted that botanical extracts often contain myriad phytoconstituents; therefore, complete characterization may be impossible for each component, and judgment of experts must be used to ensure sufficient characterization for safety and efficacy assessment.

### Probiotic characterization

Current Food and Drug Administration (FDA) labeling guidance calls for dietary supplement probiotics labels to list only the weight of the product’s total cellular mass, consisting of both live and dead microorganisms [15]. However, efficacy requires more complete probiotic characterization, including genus, species, subspecies, and strain [16]. Furthermore, because *live* organisms may be required to convey potential health benefits, a measure of CFUs (a measure of viable cells capable of replicating) should also be determined, with attention given to shelf life, as the viable microorganisms will change according to storage conditions.

### Final product form

The final forms of dietary supplements may be powders, tablets, capsules, softgels, gummies, oils, liquids, or tinctures containing the bioactive constituents in various physical (e.g., nanoparticles) and chemical formulations. Distinct dosage forms affect performance attributes, such as disintegration and dissolution, stability, and absorption, distribution, metabolism, and excretion (ADME). Thus, the form must be considered in data review and specified in use guidance. To ensure that variation due to matrix does not extend beyond typical interindividual variation, to the extent that the association between the health benefit and the active ingredient, constituent(s), principal ingredient, or metabolite profile is known, the matrix effect on that association or markers should be established.

### Disintegration and dissolution

Disintegration and dissolution are 2 characteristics of supplements that have a major impact on supplement performance. Disintegration is the process by which a supplement form (tablet, capsule, etc.) breaks down into smaller particles. Dissolution is the process by which the components of a supplement dissolve into solution. A consideration for supplements in pill form (e.g., tablet or softgel) is the ability for the whole product to disintegrate and subsequently for the bioactive constituents to dissolve for intestinal absorption and bioaccessability. Testing of products has demonstrated that disintegration and dissolution cannot be assumed [17]. Formulations, coatings, fillers, other excipients, and shelf storage (allowing time for gelatin cross-linking and other changes) all affect product disintegration rates [18,19]. Once a pill has disintegrated, the target ingredient’s form may affect dissolution and thus absorption. Characterizing disintegration and dissolution is an important factor for evaluating the potential safety and efficacy of a given supplement form. As an example, a plant sterol supplement in crystalline form is less effective for lowering LDL cholesterol than a plant sterol in a lecithin emulsion [20]. Dissolution is more difficult and expensive to test than disintegration as it requires specialized instruments and methods to measure the bioactive in solution. Ideally, dissolution studies should be conducted in biorelevant media (i.e., fasted state simulated gastric fluid, fed state simulated gastric fluid, fasted state simulated intestinal fluid or fed state simulated intestinal fluid). Thus, detailed dissolution data for a specific supplement product may not be available. Nonetheless, consideration of chemical form and review of the literature can be enough to draw some conclusions about potential for dissolution (as with the example of plant sterols in crystalline compared with emulsion form). United States Pharmacopeia provides standards for disintegration and dissolution testing, including specific equipment, methodology, quality control, and interpretation. These resources should be used by manufacturers to conduct testing and are useful in reviewing dissolution and disintegration testing.

### Terminology

It should be recognized that there is a lack of standardization in nomenclature, and that terminology is often field-specific [21]. Therefore, clear and unambiguous terminology is needed throughout every step of research, data review, or development of use guidance. Specificity is needed for describing a dietary supplement and its ingredients, and descriptive terms should be clear and unambiguous. Descriptive terms can have different levels of specificity. For example, a botanical supplement labeled as Ashwagandha might contain root, seeds, flowers, leaves, or a combination of plant parts [22]. The different parts of the Ashwagandha plant have different compounds and different purported biological activities [22]. Similarly, vitamin E supplements are available as different isoforms, as well as stereoisomers, all of which have different biological activities and ADME characteristics [23]. As another example, the term cinnamon refers to a genus; further specification of species conveys important information about expected chemical composition, and thus its biological activity [24]. Lack of harmonization in terminology, especially for plant-derived components in nutrition and related research, innovation, and regulatory efforts, is an impediment to clear communication, and suggestions for improving harmonization of terms and nomenclature have been discussed in detail by Frank et al. [21].

## Step 2: Evaluate Safety

**FIGURE 3:**
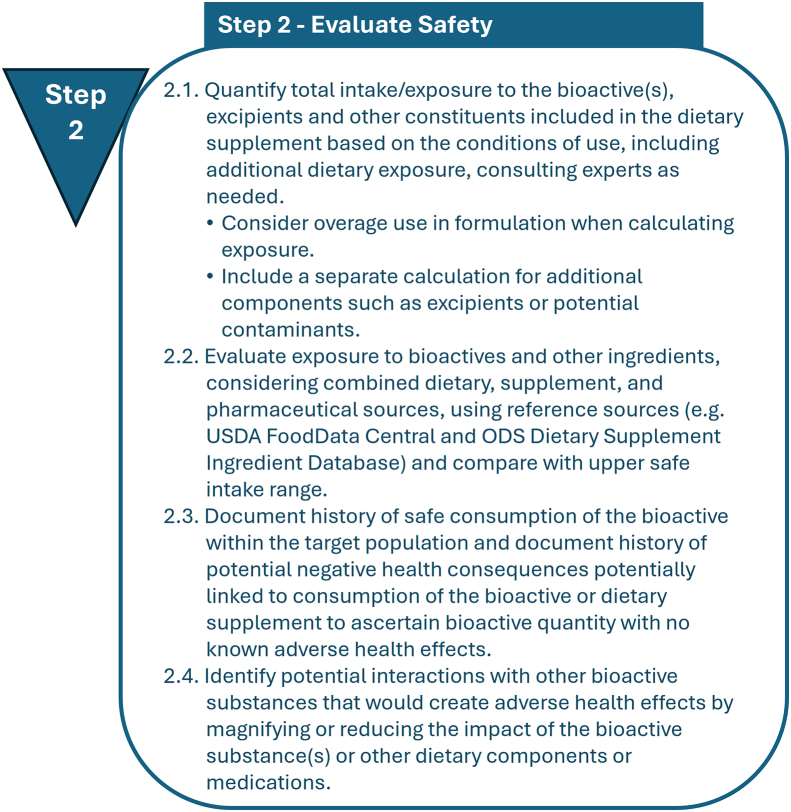
Steps for evaluating the safety of a dietary supplement bioactive in the context of other features of the dietary supplement for the development of use guidance. ODS, Office of Dietary Supplements.

Safety is a critical component for progressing toward a recommendation for non-nutrient bioactive–containing dietary supplement use. When evaluating risk, lack of evidence demonstrating risk does not necessarily imply lack of risk. Safety evaluation for bioactive constituents in foods is addressed in the paper by Yates et al. [10], and here are provided additional perspectives related to bioactive-containing dietary supplements (Figure 3).

Evidence related to safety should be supported by studies of ADME, biomarkers of adverse effects, toxicology, risk assessment, and biostatistics. Useful guidance for safety evaluation of supplements and the associated complexities can be found in the various guidance documents of the FDA and in other recent authoritative publications [11]. Evidence to be evaluated should include all available data and can include human data [randomized clinical trials (RCTs), epidemiological studies, adverse event case reports, and history of use information], relevant animal research, and in vitro studies. All available data pertaining to adverse events should be considered. Information on analogs and related substances can provide additional insights. Data from these categories should be integrated into a larger picture of potential risk. Integration of data can confirm biological plausibility and highlight gaps and inconsistencies. If a supplement is reformulated, the risk should be reassessed because reformulation changes may result in different substances present in the product as well as differences in ingredient absorption, bioavailability, distribution, metabolism, and excretion.

Dietary supplements present a unique set of conditions of use that impact constituent exposure and product safety assessment compared with that of conventional foods. These include the dose and purity of the bioactive(s) (e.g., dried plant powder compared with plant extract compared with isolated compound), product matrix (tablet, capsule, liquid, w/ or w/o excipients); target/intended population, recommended serving size, frequency and duration; recommended timing and use conditions (e.g., fed compared with fasted; avoiding certain medications or foods, etc.). All these factors impact the exposure and ADME of bioactive(s) and other constituents in dietary supplements in a manner that is distinct from conventional foods.

### Safety and dose

With non-nutrient bioactive–containing dietary supplements, dose is a particular safety risk, because supplement form and matrix can increase the exposure of a substance (either through increased bioavailability or potential overconsumption), as compared with food sources that tend to limit exposure due to their volume, other constituents, and energy-providing nutrients. NASEM developed a risk assessment model for nutrients to set tolerable upper intake levels (UL) for nutrients [25]. Setting a UL involves review of toxicity data, selection of critical effects, evaluation of dose-response, and assessment of uncertainty. The UL is determined for substances with known adverse effects by identifying the lowest observed adverse effect level (LOAEL) or the no observed adverse effect level (NOAEL), then dividing by an uncertainty factor (UF) based on the robustness of the data. UFs consider interindividual variability in response, extrapolations from animal models to humans, extrapolations of subchronic exposures to chronic exposures, and incomplete data. ULs have been set for 24 of the 37 vitamins and minerals that have been reviewed.

To date, no ULs have been set in the United States for nonessential bioactives, although the need has been recognized [26]. The UL approach has limited utility when there is a lack of toxicity data and lack of an established adverse effect, without which a LOAEL and a NOAEL, thus subsequently a UL, cannot be set. An alternative approach, termed either the *highest observed intake* (HOI) or the *observed safe level* (OSL), has been proposed and may be useful until more authoritative guidance is available. The HOI and OSL are 2 terms for the same concept, which is the highest intake with convincing evidence of safety based on evidence of acceptable quality, even if there are no established adverse effects at any level [[27], [28], [29]]. HOI is the term adopted by the WHO and FAO [28]. The suggested approach is to screen available data to identify a critical effect (i.e., an observable hazard). If a critical effect is identified, then 1 should proceed with the UL approach. If no critical effect is identified, then the data are further screened to determine the highest intake for which the data demonstrate that the occurrence of a critical effect or hazard is convincingly excluded. In the absence of established safe upper intake levels for a bioactive substance, experts in risk assessment will need to evaluate the data with consideration of not only intake from the supplement under review, but also intake from other dietary or pharmaceutical sources of the bioactive(s) in the supplement.

An additional consideration for assessing safe dosage protocols is product overage. Overage, which refers to the inclusion of higher amounts of an ingredient over the label value during formulation, is permitted by the FDA to ensure that the supplement contains at least the label amount throughout the product’s shelf life, a requirement by law. The specific amount of overage varies among supplements and can depend on the active compound, processing methods, and shipping and storage conditions. Ideally, the potential for overage should be considered in a recommendation such that the ingested amount will not exceed safe upper limits. However, it is recognized that information on overage may be proprietary and difficult to obtain.

#### Supplement contamination

Both foods and supplements can be naturally contaminated with harmful substances, such as heavy metals or pesticides from agronomic treatment. Practices as simple as irrigation with low-quality water can introduce heavy metals into soil and crops [30]. However, the differences in processing, use, or form can result in very different exposures to contaminants depending on source (food compared with supplements) [31], with supplements having potential for particularly high exposure when in concentrated form. The potential for contaminant exposure must be considered in the development of guidance, keeping in mind achievable quantities for ingestion and potential exposure to harmful substances, including the possibility of use of multiple supplements thus resulting in additive exposure to contaminants.

#### Supplement adulteration

Product adulteration can occur for food products as well as dietary supplements, though the approaches to mitigating the negative effects of adulteration differ between food and dietary supplements. Adulteration may be the result of either intentional or unintentional inclusion of undeclared ingredients in a final product. Intentional adulteration falls into 2 categories: economic adulteration and pharmaceutical adulteration [32].

Economically motivated adulteration can occur for both food or dietary supplements and occurs when a product supplier adds an undeclared ingredient to a supplement for economic gain or replaces the declared ingredient with a cheaper, undeclared ingredient. For example, true cinnamon (*Cinnamon verum*), which is consumed for purported but not well-confirmed glucoregulatory benefits, has been found to be adulterated with a cheaper, lower quality alternative *Cinnamon cassia*. Such a dilution or replacement of true cinnamon with the lower quality alternative presents a potential health risk in that *Cinnamon cassia* contains a much higher content of coumarin, elevating the risk for hepatotoxicity [33]. Alternatively, adulterants might be added to increase the weight of a product so that suppliers can charge more for a given amount of product.

Another reason for intentional adulteration is the enhancement of product efficacy with active drugs. This type of adulteration is termed pharmaceutical adulteration. Weight loss and sexual enhancement products are particularly prone to this type of adulteration [34,35]. Such cases can include adulteration with substances that are not permitted for inclusion in supplements, either because they are pharmaceuticals, thus requiring a prescription, or because they have been removed from the market due to safety concerns [34], and these adulterants pose safety concerns because they can increase cardiac, central nervous system, and other health risks [31].

The potential for adulteration and the associated health risks should be evaluated and addressed in creating intake or use guidance. Economically driven adulteration may introduce less controlled ingredients into a product, making it more difficult to assess overall safety risk, whereas pharmaceutical adulteration for the enhancement of products may directly contribute to or amplify health risk. Although it can be difficult to determine which supplements or products have been adulterated, it is important to recognize which ingredients are more susceptible to adulteration and to communicate this risk when making recommendations.

#### Excipients

Excipients are substances used in the formulation of dietary supplements to support or enhance the final form of the product. Excipients can include binders, fillers, emulsifiers, colorants, flavors, coatings, or preservatives [36]. Potential risk and/or effects of excipients should be considered from a safety standpoint, such as the potential introduction of allergens or modification of stability or delivery properties of the bioactive.

#### Product shelf stability

A product’s vulnerability to degradation during normal manufacturing, storage, shipment, and end-use by the consumer is an important consideration for product quality and safety. Certain oil-based products are especially vulnerable to oxidation during shelf storage. For example, long-chain PUFAs, such as omega-3 fatty acids in fish oil supplements, are particularly susceptible to oxidation. Studies have shown that fish oil supplements contain as much as 62% oxidized lipid, which can have detrimental health effects [37]. In food, compromised quality related to shelf life is often detectable by sensory evaluation (smell, taste) alone. Without sensory indications to detect loss of product integrity, other approaches are needed. Shelf stability may also affect products sold in certain forms, such as tablets compared with soft gels. During storage, temperature and humidity can alter the water content of softgel capsules, which impacts the hardness and adhesion of film coatings as well as cross-linking [38]. Cross-linking can occur between gelatin molecules or between a gelatin molecule and interior components, reducing dissolution. Factors affecting product stability and integrity include temperature and other storage conditions, exposure to moisture, light, or oxygen, and can be modified by the primary packaging system used. Ingredient and final product stability information may be difficult to obtain, but ideally should be considered if possible, and reports of problems with product integrity should be a factor in the assessment of product safety.

#### Interactions of supplement bioactives with food, nutrients, medications, or other supplements

Just as food and medications can interact, the classic example being medication-grapefruit interactions, coingestion of supplement bioactives with food, medications, or other supplements should be considered for intake guidance. Ingestion of supplement bioactives in the fasted compared with the fed state can influence bioavailability, biological activity, and/or potential adverse effects. For example, risk for hepatotoxicity is increased when green tea extract is consumed in the fasted state compared with the fed state, and coingestion with caffeine affects risk as well [[39], [40], [41], [42]].

Supplement bioactives can also influence nutrient absorption [42], such as the case of catechins in green tea extract which bind to iron, reducing iron absorption and status [43,44]. St. John’s wort, a particularly popular supplement for antidepressant effects, induces a specific xenobiotic enzyme (CYP3A4) that is a pathway for metabolism of many drugs and thus conveys a relatively high risk for supplement–drug interactions [45]. Documented adverse interactions between St. John’s wort and medications include organ transplant rejection (due to reduced blood levels of an immunosuppressant) [46] and suppressed efficacy of oral contraceptives [47] among others. The potential for interactions must be thoroughly considered, and a more thorough discussion of the evaluation of potential for interactions can be found in the publications by Gurley [5,48,49]. Proper use of the supplement bioactives to minimize potential side effects should always be clearly specified when making a recommendation.

#### Population-targeted considerations

Vulnerable populations at particular health risk are an important aspect for conditions of use and safety. Preterm infants, for example, are a vulnerable group with respect to probiotics [50]. A supplement that is safe to use on one group of people may pose serious health risks to a different population. If a supplement has not been evaluated in a vulnerable population, it should not be recommended for that population without consultation with their physician or a healthcare professional with suitable experience in evaluating safe use.

#### Postmarket surveillance and pharmacovigilance

For bioactive substances, as with all foods, drugs, and supplements, pharmacovigilance is an important safety consideration. Pharmacovigilance is defined by the WHO as the activities related to the detection, assessment, understanding, and prevention of adverse effects or other medicine- or vaccine-related problems [51]. It plays a crucial role in ensuring the safety of medications and protecting the health of patients because it mostly focuses on the identification of potential adverse drug reactions (ADRs) after medicinal products have been licensed and released to the public.

A form of pharmacovigilance, collection, monitoring, and reporting of adverse events to FDA is required for dietary supplements in the United States. FDA has provided detailed guidance outlining how dietary supplement firms can meet these requirements. The effectiveness of this requirement could be improved by enhancing the ability to recognize trends more quickly and appropriately through existing data sources, perhaps by applying artificial intelligence (AI) technologies [52].

Key aspects of pharmacovigilance [53]:

• Detection and assessment: identifying and evaluating unexpected or known harmful reactions (ADRs) and other issues.
• Understanding: analyzing data to grasp why these problems occur and their impact on patients.
• Prevention: implementing measures to minimize harm, such as updating warnings, changing usage guidelines, or even withdrawing drugs if necessary.
• Postmarket surveillance: continuing surveillance long after a drug is approved, monitoring its effects in diverse populations.
These key elements are important considerations when evaluating the safety of a bioactive supplements.

### Sources of safety data

Useful safety information can be obtained from human trials or history of use reports, animal studies, and in vitro data.

#### RCTs

Considerable weight can be given to properly powered, high-quality RCTs that incorporate safety-related outcome measures [54] or epidemiological cohort studies designed to detect adverse effects of dietary supplements [55]. Double-blinded RCTs are the reference standard of scientific evidence for humans, but RCTs are usually designed to test efficacy rather than safety. Although adverse events must be reported during a clinical trial, the trials themselves are usually small in the number of subjects and relatively short in duration, and thus lack of observed adverse events during a clinical trial is not necessarily an assurance of safety for longer-term use in a broader population. RCTs also generally fail to capture interactions with other supplements, foods, or medications. Sorkin et al. [13] discussed similar issues for RCTs of natural products.

#### Observational/epidemiological study data

A particular vulnerability with observational epidemiological studies is that the dietary supplements under study are often poorly characterized [49], which increases variability in response and limits the usefulness of the resulting data. Furthermore, epidemiological studies generally include highly focused or limited health outcome measures. Harm can be broad and endpoints in humans are often unknown. Therefore, adverse effects can easily be missed if the harm is not captured by an outcome measure. Sorkin et al. [13] discussed similar issues for studies of natural products.

#### Animal study data

Data from animal studies should be considered for evaluating possible harm. Evidence of harm from animal studies is often indicative of potential for human harm, even if human data do not exist. Toxicity should be addressed by multiple techniques to generate a robust body of preclinical data on safety and efficacy. Preclinical studies should be followed by carefully designed human research to confirm safety and efficacy [54]. In vitro evidence of harm can be an independent indicator of risk if the in vitro model correlates with an adverse health event in humans or animals [56].

#### History of use data

History of use data may be helpful when evaluating the body of evidence for the supplement or its constituents. Formal reports in ethnobotanical and ethnomedical literature should be the source of history of use information. Traditional cautions for contraindications on botanical use, vulnerable populations, or uses leading to potential adverse events can be helpful in determining areas for concern. Such negative information is even more useful when there is support from in vitro or animal data to confirm the adverse effects. But it is important to consider the relevance of the data with respect to the constituent or product’s specific form (including part of the plant used), dose, length of use, the population, and conditions of use. History of use data is often with a food form which is different from a purified form or the constituent in a dietary supplement. It should additionally be noted that bioactives in historical use reports are not likely well characterized.

#### Adverse event reports

The United States FDA maintains the Human Foods Complaint System, which is a postmarket surveillance system for products regulated under the FDA’s Human Foods Program, including dietary supplements. In accord with the Dietary Supplement and Nonprescription Drug Consumer Protection Act of 2006, dietary supplement firms are required to report to FDA all serious adverse events within 15 d of receipt and maintain records of all adverse events received from consumers. The adverse events received by FDA are entered into the Human Foods Complaint System. In addition to these mandatory adverse event reports submitted from industry, The Human Foods Complaint System database also includes adverse event reports from voluntary reporting from industry, consumers, and healthcare practitioners. Raw data can be downloaded for review. It is possible that negative side effects associated with over-the-counter products may be underreported as patients may not disclose their use of supplements or related adverse events to healthcare providers, who are the primary reporters of such events. Credible reports of adverse effects in humans must be evaluated, and a judgment must be made with respect to the likely cause of the adverse event and whether it was related to the supplement or ingredient. Credible reports of adverse events related to a dietary supplement should be noted and evaluated, but the final judgment must be made on the body of evidence as a whole.

#### New approach methodologies in toxicology

New approach methodologies (NAMs), as applied in the field of toxicology, are new and emerging technologies designed to enhance risk assessment and better address critical information gaps while reducing the use of animal models. These approaches include techniques such as in vitro organoid models, compilation of multiomics data, and computational methodologies. Efforts are underway to standardize these approaches and to improve their translation to human risk assessment. Because these approaches continue to develop, they will provide new insights into risk assessment of chemicals including bioactive substances. Further discussion of NAMs in toxicology can be found in Deepika et al. [57].

## Step 3: Conduct Efficacy Review

**FIGURE 4:**
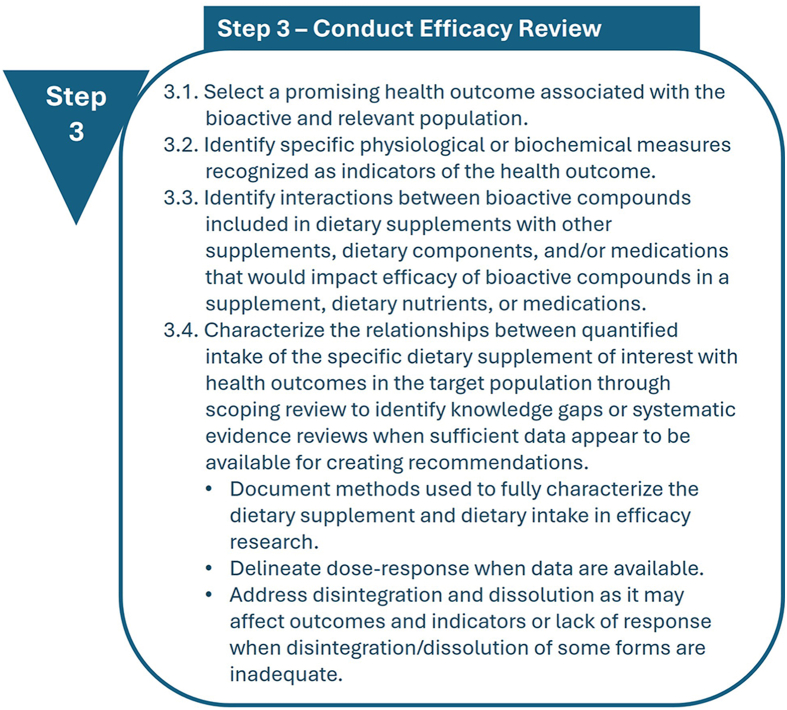
Steps for assessing the efficacy of a dietary supplement bioactive in the context of other features of the dietary supplement for the development of use guidance.

Efficacy evaluation for food bioactives and non-nutrient bioactive–containing dietary supplements is very similar (Figure 4). Conducting an efficacy review for food bioactives is described in detail in the paper by Yates et al. [10]. An additional consideration for dietary supplement bioactives is the increased potential for interactions, which may affect efficacy, although interactions can occur for bioactives in food as well. Different levels of confidence in potential health benefits may be justifiable if the risk is low. Nevertheless, evaluation of efficacy data is complex [11] and should include experts in nutrition, physiology of the health-related pathways under review, ADME, biostatistics, and Evidence to Decision frameworks. Evidence to be evaluated should include all available data and should include in silico screenings, in vitro studies, animal studies, and human data (RCTs, epidemiological studies, and history of use information).

### Efficacy considerations

#### Indicators of efficacy

Efficacy should be evaluated by a biomarker that is widely accepted by the research community for the target health outcome. Indicators of efficacy are varied and should reflect changes in metabolism, general health, performance, or reduction of disease risk, as accepted by authoritative health professional groups or organizations. Indicators of efficacy are strongest if they are validated surrogate markers for disease risk, morbidity, mortality, performance, or impact on essential physiologic functions. Dose-response data demonstrating that a greater exposure to the bioactive results in a more pronounced change in the biomarker indicator is desirable. Changes in blood concentrations of consumed bioactives are not necessarily evidence of physiological functionality.

#### Product form and efficacy

It is important that the product form under review is well characterized, defined, and understood, as described above. Efficacy evaluation of a poorly characterized product could lead to spurious conclusions. Furthermore, it is important to ensure that disintegration and dissolution have been addressed and confirmed for the forms under study, as null results for efficacy can result from poor disintegration and dissolution of a dietary supplement form rather than a lack of efficacy of a particular bioactive. Note that research on purified compounds may not necessarily translate to similar outcomes with *products* containing the same compound, because formulation, matrix, and excipients will affect the absorption and metabolism of the compound of interest. Although it may not be possible to establish a reasonable causal link between a bioactive and a health outcome, it is preferable for the purported beneficial compound(s) to be indicated for a given supplement product.

#### Interactions that may influence efficacy

Interactions between dietary supplement bioactives and other dietary components or medications that affect efficacy should be identified. Interactions among dietary components may increase or decrease efficacy of a given bioactive, and such interactions should be considered with respect to dosage recommendations and conditions of use. Conversely, the effect of the bioactive on efficacy of nutrients or medications should also be considered.

### Sources of efficacy data

#### RCTs

The reference standard of scientific evidence for humans is the properly designed and conducted RCT [54]. Null results from an RCT may not necessarily indicate a lack of bioactivity, if bioactivity is limited by a low dose, insufficient intervention time, inadequate disintegration or dissolution, or insufficient statistical power (usually because of low subject numbers). In addition, RCTs often are not powered to detect differences in response related to ethnic diversity, sex, and other phenotypic characteristics. Many trials have controlled conditions or diets, and so they will not test all interactions that may affect efficacy. With RCTs, the *quality* of the study design and execution is more important than *quantity*; therefore, multiple poorly designed studies do not override evidence from a single, well-designed study. An RCT should be sufficiently powered, include a control group, be properly randomized, have double-blinded treatments if possible, use well-characterized treatment materials, and produce statistically significant and clinically meaningful results. There is no clear minimum number of RCTs that constitute convincing evidence; however, replication of findings in independent labs contributes to confidence in the overall results. Epidemiological studies suffer from the limitation that products are often poorly characterized [55], and thus, the lack of effect may be in part related to variability in response among study subjects resulting from variability in product composition, form, dissolution/disintegration, or other factors.

#### Animal and in vitro data

Considerations for translational relevance of animal or in vitro data are discussed in Sorkin et al. [13] in the context of natural products, and many of those principles apply to dietary supplement bioactives. Animal data can contribute to the body of evidence, but it is important that the animal model is an appropriate model for the bioactive class, outcome variable, and mechanism of action. For example, rats and mice are sometimes used for studies of cholesterol-lowering agents, but rats and mice lack cholesterol ester transfer protein (an important aspect of cholesterol trafficking in humans), thus are poor models for human cholesterol metabolism [58]. Similarly, rodents are a poor model for the effect of lutein and zeaxanthin on age-related macular degeneration, as rodents lack a macula. This anatomical difference limits the translation of rodent studies on lutein/zeaxanthin and macular pigment [59]. Other limitations of the translatability of animal studies to humans include interspecies differences in xenobiotic metabolism and failure to consider allometric scaling when comparing doses among species [[60], [61], [62]], and these factors should be evaluated for animal studies under consideration. Animal studies should also include both male and female animals because responses can be different by sex [63]. It should be noted that administration by gavage is not identical to standard oral ingestion of a capsule, gummy, or other supplement form, thus bioavailability of components by those 2 administration methods might differ. In vitro evidence can also provide augmentation of the plausibility of efficacy of ingredients used in supplements.

#### History of use data

History of use evidence must also be evaluated with careful consideration of form. History of use data is often based on food intake, which may be very different from use of a supplement. For example, much historical evidence on traditional medicine uses of mangosteen is based on consumption of the palatable fruit or its dark purple peel [64]. In contrast, mangosteen supplements are commonly made from the mangosteen pericarp, which is not generally eaten. Thus, historical evidence for mangosteen health benefits may be irrelevant to most mangosteen supplements, as may occur with other dietary supplements as well. History of use evidence must be from reliable sources such as peer-reviewed ethnobotanical literature rather than anecdotal evidence. Good practices for translation of evidence from ethnobotanical observations of natural products are discussed in Sorkin et al. [13] and are relevant to compounds found in dietary supplement products.

#### NAMs for efficacy evaluation

As with toxicology, NAMs offer great promise to accelerate nutrition efficacy research and enhance the body of evidence for the role of bioactives in the promotion of health. New tools, such as 3D cell models, organoid-on-a-chip systems, multiorgan in vitro systems, and computational methods to analyze multiomic data sets, offer promise for new insights into understanding of bioactive molecular and physiologic mechanisms. Although many of these approaches still have limitations, efforts are ongoing to overcome hurdles so that these methods can be increasingly used to augment the body of research [65].

## Step 4: Decide on Summary or Recommendation

**FIGURE 5:**
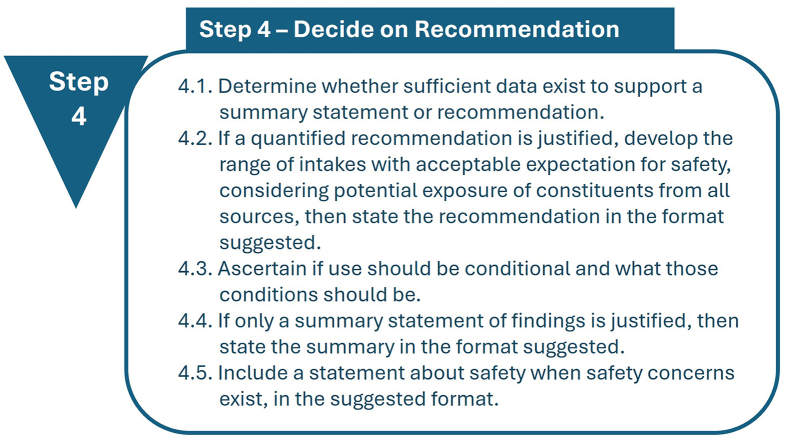
Steps for creating guidance statements based on findings from the previous steps of the framework for development of use guidance for non-nutrient bioactives in dietary supplements.

Once the evidence has been collected, review and interpretation are needed to decide on an appropriate recommendation (Figure 5). The recommendations presented in this section are intended to complement the framework previously described for food, as the same principles apply.

### Review and synthesis of efficacy and safety research: scoping or systematic review

One of the first decisions to be made in review and synthesis of the data is whether the body of evidence is more appropriate for a scoping review compared with a full systematic review. A scoping review is used to summarize research and identify gaps, whereas a systematic review is appropriate to create recommendations for use. Given the likelihood of scant research on some bioactive substances, scoping reviews may be the first logical step for them.

**FIGURE 6:**
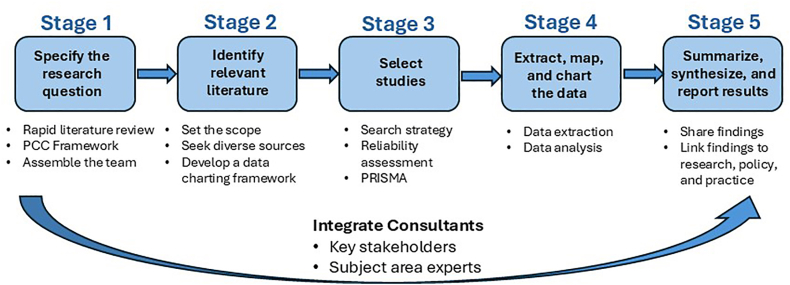
Schematic of the data review process for evaluating the body of evidence related to efficacy and potential safety issues related to non-nutrient bioactives in dietary supplements. PCC, Population, Concept, and Context.

Scoping activity is a form of knowledge synthesis, and scoping reviews aim to describe the breadth of an existing knowledge base and inform future research, practice, and policy. Often, a scoping review may be done before undertaking a systematic review, to determine if the body of evidence appears to justify a systematic review. If the body of evidence collected in the scoping review supports moving to a systematic review, then a systematic review should also be undertaken to make recommendations for use [66,67]. The scoping review steps are shown in Figure 6.

There are similarities in the rigor of scoping and systematic reviews with respect to the steps for specification of the research question (stage 1) and identification of relevant research (stage 2). With non-nutrient bioactive–containing dietary supplements, the considerations presented in both the safety and efficacy steps above should be included in stages 1 and 2. Study selection (stage 3) may be broader and include as much research as possible, as long as the data charting clearly identifies shortcomings of existing research and/or characterization of the bioactive-containing supplement. Extracting, mapping, and charting the data (stage 4) does not include appraising the quality of the studies, but can record the presence or absence of documentation for key attributes important for both safety and efficacy evaluations. The final step is to summarize, synthesize, and report results (stage 5). This is likely to focus on gaps identified, future research, and links to policy and practice that need to be addressed. Another key difference from the scoping review is the incorporation of expert input (outside of the original team) during stage 5 and further description of the potential impact of missing data or research methods that need improvement.

Caution is recommended for use of or reference to systematic reviews for reports of harm. Although guidelines for systematic review methodology recommend that systematic reviews address both benefits and harms to provide a balanced evaluation of data, the methodology to assess benefits in a systematic review is not necessarily appropriate for the assessment of harm. Application of methods for systematic review of benefits to identify and synthesize potential harm-related evidence is inadequate and misleading, underestimating potential harm [68]. In addition, systematic reviews generally draw information from randomized controlled trials that were designed to evaluate benefit rather than harm. Systematic reviews of harm should *1*) specifically address harm (rather than include harm as an auxiliary aspect to a review of benefit), *2*) assess all important harms when possible, *3*) ensure that studies include large sample sizes, diverse populations, and long duration, *4*) include observational data, *5*) specify details of analyses and key assumptions, *6*) describe handling of rare events and missing data, and *7*) disclose limitations [68,69]. Assessment of harms requires understanding of terminology to describe different types of harms, classification of type and frequency of harm events, evaluation of severity, and adequate detection of harm events [69]. Until there is a paradigm shift in the approach to systematic review of data on harm, harm evaluation as an aspect of a systematic review should be regarded as potentially missing important risk information.

### Final framework and proposed template statements for intake and use guidance for non-nutrient bioactive–containing dietary supplements

**FIGURE 7:**
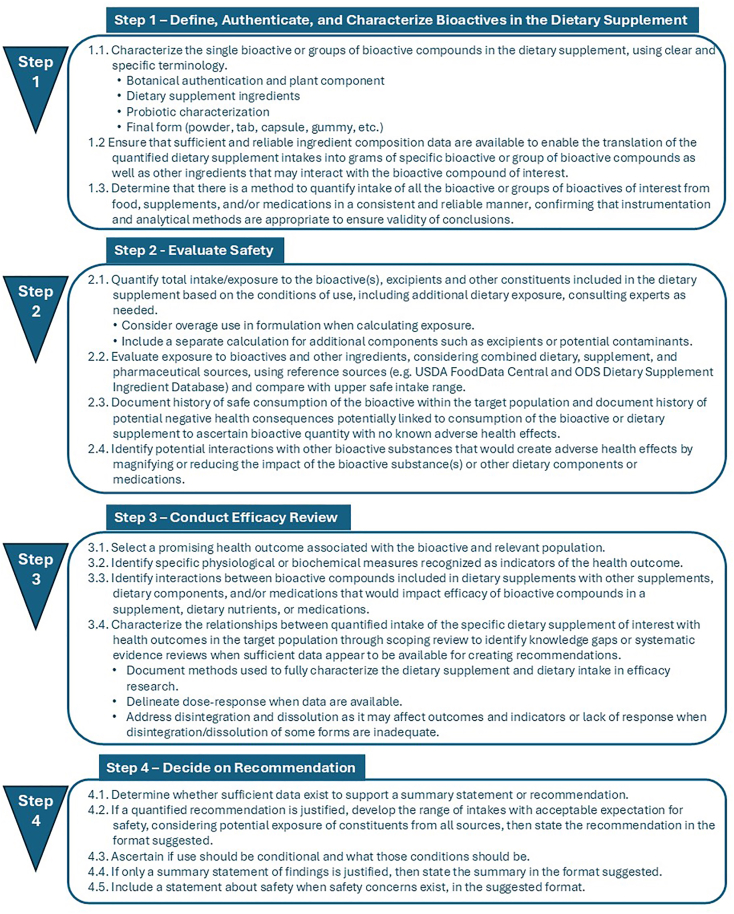
Stepwise framework for developing intake or use guidance for non-nutrient bioactives in dietary supplement form. The steps of this framework should be completed sequentially. On completion of step 4, a decision is made on the format of the use guidance. Templates for different use guidance statements are shown in Figure 8. The selection of the format for the guidance statement will be driven by the findings from the first 3 steps of this framework. ODS, Office of Dietary Supplements.

The adaptations delineated above to the structure of the previous framework for development of intake recommendations for food bioactives allow extension of the framework to encompass many of the specific considerations for bioactives in dietary supplement form. The modified framework adapted for development of guidance for use of non-nutrient bioactive–containing dietary supplements is shown in Figure 7. The steps facilitate the collection and interpretation of the necessary information to create summary statements and/or use recommendations.

### Drafting of summary statement or use recommendation

**FIGURE 8:**
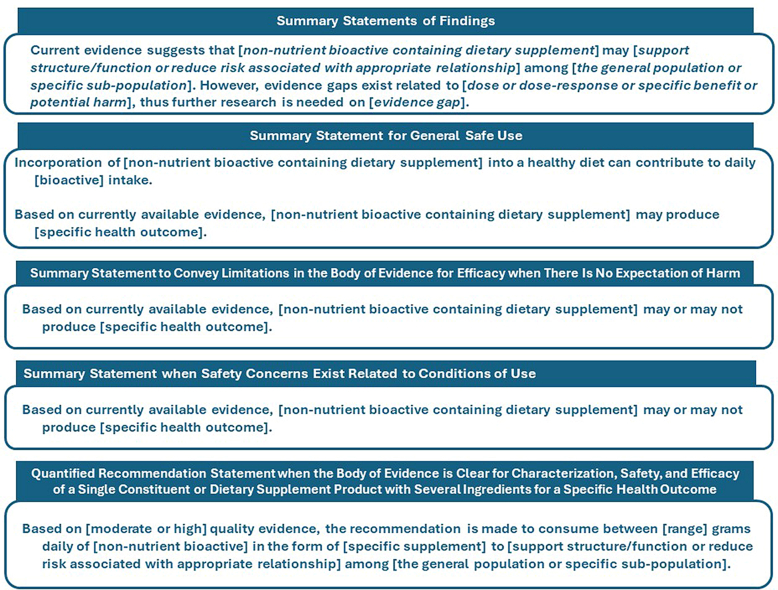
Sample templates for summary statements or recommendations.

Dietary supplements are a very diverse group of products. Some dietary supplements are minimally altered plant components, such as dehydrated apple powder which maintains many compositional characteristics of an apple, whereas other products are synthesized or purified compounds, complex botanical extracts, or proprietary blends of ingredients. Because of this diversity, a single recommendation format would not be applicable to cover the vast array of products on the market. The characteristics of the dietary supplement product and the body of evidence will determine what type of recommendation is appropriate. The suggested format for different summary or recommendation statements is shown in Figure 8.

#### Summary statement of findings

In some cases, a summary statement of findings may be most appropriate. A summary statement would be particularly applicable for cases in which evidence is suggestive of benefits, but insufficient to create a recommendation statement. The summary statement would state the possible benefit and highlight gaps in knowledge. The suggested format is as follows: “Current evidence suggests that (*non*-*nutrient bioactive–containing dietary supplement*) may (*support structure/function or reduce risk associated with appropriate relationship*) among (*the general population or specific subpopulation*). However, evidence gaps exist related to (*dose or dose*-*response or specific benefit or potential harm*), thus further research is needed on (*evidence gap*).” Such a statement would be useful for health professionals in advising patients and clients, for individuals seeking to improve their health with use of dietary supplements, and for researchers aiming to fill knowledge gaps.

#### Summary statement for general safe use

In some cases, a summary statement for safe use may be most appropriate, to provide context for use of a non-nutrient bioactive–containing dietary supplement to contribute to bioactive intake or to achieve a health benefit. The suggested format for a statement for contributing to bioactive intake is as follows: “Incorporation of (*non*-*nutrient bioactive–containing dietary supplement*) into a healthy diet can contribute to daily (*bioactive*) intake.” A general use statement related to a health benefit could be formatted as follows: “Based on currently available evidence, (*non*-*nutrient bioactive–containing dietary supplement*) may produce (*specific health outcome*).”

#### Summary statement to convey limitations in the body of evidence for efficacy when there is no expectation of harm

In some cases, the body of evidence may not strongly support a health benefit, whereas use is unlikely to cause harm. In such cases, a summary statement could be issued to reflect the body of evidence. For example, if the data neither support nor deny a health outcome, a summary statement may be issued as follows: “Based on currently available evidence, (*non*-*nutrient bioactive–containing dietary supplement*) may or may not produce (*specific health outcome*)” and an additional statement about safety could be included.

#### Summary statement when safety concerns exist related to conditions of use

If safety concerns exist related to conditions of use, an additional statement should be included in the recommendation in the following form: “The (*non*-*nutrient bioactive–containing dietary supplement*) should/should not be (*consumed*) under (*these conditions of use*).”

#### Quantified recommendation statement when the body of evidence is clear for characterization, safety, and efficacy of a non-nutrient bioactive in the form of a dietary supplement, as a single ingredient or with several ingredients, for a specific health outcome

In other cases, when the body of evidence for clear characterization, safety, and efficacy for a specific health goal is deemed sufficient for a quantified recommendation, then the recommendation in the format presented by Yates et al. [10] may be warranted: “Based on (*moderate* or *high*) quality evidence, the recommendation is made to consume between (*range*) grams daily of (*non*-*nutrient bioactive[s]*) in the form of (*specific supplement*) to (*support structure/function* or *reduce risk associated with appropriate relationship*) among (*the general population* or *specific subpopulation*).”

### Postrecommendation considerations

The proposed guidance statements represent an important step toward safer, more effective, and appropriate use of bioactive dietary supplements. However, a use or guidance statement should not be viewed as an endpoint. To ensure long-term credibility, utility, and impact of the guidance, continued efforts from both the research community and industry will be essential. Ongoing evaluation of postmarket surveillance systems is warranted to strengthen the detection and assessment of adverse effects associated with bioactive supplements. Additionally, the successful implementation of use and guidance statements will depend on their adoption by healthcare practitioners. Targeted educational initiatives and professional outreach or training will facilitate equipping practitioners with the knowledge and confidence needed to appropriately interpret and apply the recommendations in clinical practice.

Dietary Supplements can support health in myriad ways. Given the large number of diverse supplements on the market and the demonstrated interest from individuals in using dietary supplements to support health, it is important that the evidence for safe and efficacious use of supplements be clearly communicated to consumers. Summary statements or quantified recommendations for use should be made available to consumers and the community of health professionals when supported by the body of evidence.

The stepwise framework described here for non-nutrient bioactive–containing dietary supplements extends the previous framework for developing recommendations for bioactive constituents in food to include special considerations for dietary supplements. The potential health benefits and safety concerns are similar for bioactives in both foods and dietary supplements; however, the supplement forms and conditions of use differ from foods in exposure risk, thus intake or recommendations for use need to take these differences into account to ensure efficacy and safety. These resulting summaries and recommendations may serve to: *1*) inform scientists about critical study design features and knowledge gaps, *2*) guide industry about important issues that need to be addressed in dietary supplement development and production, *3*) inform project or grant reviewers and evaluators of critical details for robust study designs, and *4*) inform health professionals providing advice to individuals and the general public for the safe and efficacious use of non-nutrient bioactive–containing dietary supplements as part of a healthy diet.

## Author contributions

The authors’ responsibilities were as follows – JAN: drafted the original sections based on author meetings; JAN, EM: designed the figures; JAN, TK: finalized the text and figures; and all authors: contributed to revising drafts and approved the final manuscript.

## Funding

The work was a product of the Bioactives, Supplements, and Functional Foods research area of the International Life Sciences Institute United States and Canada. Funding for this product came from Amway, Pharmavite, and the International Life Sciences Institute of United States and Canada.

## Declaration of Generative AI and AI-assisted technologies in the writing process

The author(s) declare that no generative AI or AI-assisted technologies were used in the writing of this manuscript.

## Conflict of interest

AS is an Editorial Board Member for Advances in Nutrition and played no role in the Journal’s evaluation of the manuscript. The other authors report no conflicts of interest.
